# Ripasudil alleviated the inflammation of RPE cells by targeting the miR-136-5p/ROCK/NLRP3 pathway

**DOI:** 10.1186/s12886-020-01400-5

**Published:** 2020-04-06

**Authors:** Zhao Gao, Qiang Li, Yunda Zhang, Xiaohong Gao, Haiyan Li, Zhigang Yuan

**Affiliations:** 1 Department of Vitreoretinopathy, Shanxi Eye Hospital, No.100 Fudong Street, Xinghualing district, Taiyuan city, 030001 Shanxi province China; 2 Department of Orthopedics, Shanxi Bethune Hospital, Shanxi Academy of Medical Sciences, Taiyuan city, 030032 Shanxi province China; 3 Department of Pathology, Shanxi Eye Hospital, Taiyuan city, 030001 Shanxi province China

**Keywords:** Ripasudil, ROCK1, ROCK2, miR-136-5p, RPE cells, NLRP3

## Abstract

**Background:**

Inflammation of RPE cells led to different kinds of eye diseases and affected the normal function of the retina. Furthermore, higher levels of ROCK1 and ROCK2 induced injury of endothelial cells and many inflammatory diseases of the eyes. Ripasudil, which was used for the treatment of glaucoma, was one kind of the inhibitor of ROCK1 and ROCK2, but whether ripasudil could relieve the LPS-induced inflammation and damage of RPE cells was not clear.

**Methods:**

We used LPS to stimulate ARPE-19 cells, the RPE cell line. After that, we detected the levels of ROCK1 and ROCK2 by western-blotting after the stimulation of LPS and treatment of ripasudil. Then luciferase reporter assays were used to confirm the targeting effect of miR-136-5p on ROCK1 and ROCK2. At last, the levels of NLRP3, ASC, caspase1, IL-1β and IL-18 were detected with the western-blotting after the knockdown of miR-136-5p.

**Results:**

The levels of ROCK1, ROCK2 and miR-136-5p in ARPE-19 cells were promoted after the stimulation of LPS. After the treatment of ripasudil, the expression levels of ROCK1, ROCK2 and miR-136-5p were suppressed. The expression of ROCK1 and ROCK2 was targeted and inhibited by the miR-136-5p. The levels of inflammation related proteins NLRP3, ASC, caspase1, IL-1β and IL-18 was also inhibited after the treatment of ripasudil. However, the expression of these proteins was rescued after the knockdown of miR-136-5p.

**Conclusion:**

Ripasudil relieved the inflammatory injury of RPE cells by upregulating miR-136-5p, therefore inhibiting the expression of ROCK1, ROCK2, NLRP3, ASC, caspase1, IL-1β and IL-18.

## Background

The monolayer pigment cells between the choroid and retina were called the retinal pigment epithelium (RPE) cells. Inflammatory response and apoptosis of RPE cells were the key factors in the pathogenesis of many retinal degenerative diseases [1]. The inflammation of the RPE cells could also lead to the occurrence and development of age-related macular degeneration (AMD) and impact the normal physiological function of retina [2]. Rho-associated coiled-coil containing protein kinase (ROCK), which can be classified into two isomers: ROCK1 and ROCK2, is a serine-threonine kinase that exists inside the cells. During the pathological process, excessive activation of rock-related proteins leads to the damage of vascular endothelial, which may induce angina pectoris, ischemic stroke and other cardiovascular and cerebrovascular diseases [3]. The migration of endothelial cells was also regulated by the ROCK pathway [3–5]. ROCK1 and ROCK2 were expressed in many organs including the retina, while excessive expression of ROCK1 and ROCK2 caused many diseases in the eye, such as diabetic retinopathy and glaucoma [6, 7]. And there is also study revealed that the ROCK1 and ROCK2 are expressed in the trabecular meshwork and ciliary muscle tissues of glaucoma patients [8]. All these results indicated that the expression of ROCK1 and ROCK2 was associated with the occurrence and development of eye related disease.

Ripasudil was the inhibitor of ROCK pathway and used as a drug for the treatment of glaucoma and some other eye diseases [6]. Application of the ripasudil could reduce the intraocular pressure (IOP) of eyes [9]. In addition, ripasudil could also reduce the apoptosis of corneal endothelial cell [10]. At the same time, it could relieve the LPS-induced vascular endothelial injury by targeting the ROCK2/eNOS signaling pathway [11]. Furthermore, recent studies have confirmed that lncRNA SNHG14 promoted the occurrence and development of inflammation by inhibiting the intracellular content of miR-136-5p which could lead to the upregulation of the ROCK1 [12]. However, some researches revealed that the overexpression of miR-136-5p could promote the expression of NF-κB, thereby aggravating the damage of the inflammatory response [13]. However, whether the ripasudil could affect the inflammation and apoptosis of RPE cells by targeting the miR-136-5p/ROCK axis was unclear.

In this study, we used LPS-induced RPE cells to simulate the inflammation of retina pigment epithelium cells. Next, luciferase reporter assays were used to detect the targeting effect of miR-136-5p on the ROCK1 and ROCK2, meanwhile the levels of ROCK1 and ROCK2 were determined after the treatment of ripasudil and LPS. At last, levels of inflammatory factors and apoptosis rates of the RPE cells, in which miR-136-5p was stably knocked down, were determined after the treatment of ripasudil or the stimulation of LPS. The results from all these experiments have clarified the efficacy of miR-136-5p on the inflammatory response and apoptosis of RPE cells induced by the ripasudil.

## Methods

### Cell culture

The RPE cell line ARPE-19 was obtained from the ATCC (Manassas, VA). And these cells were cultured in the RPMI-1640 medium (Gibco, Gaithersburg, MD, USA) which was added with 10% fetal bovine serum (Gibco, USA). These cells were placed in the 37 °C humid atmosphere with 5% CO_2_. LPS and ripasudil were added in the culture medium to simulate and treat the RPE cells. The inhibitor of the miR-136-5p (Ribbio, Guangzhou China) was transfected with the lipo-2000. All the operations of the experiment were carried out according to the instructions.

### Luciferase reporter assay

1 × 10^5^ cells were plated into the 24 well plates. After the attachment of these cells, 0.1 μg reporter vector with 3’UTR-ROCK1 or mutant-3’UTR-ROCK1 (Genechem, China), 0.02 μg Renilla luciferase vector (Genechem, China) and the miR-136-5p inhibitor or the NC were contransfected into these cells. The targeting effect of miR-136-5p on the ROCK2 was detected in the same way. After 48 h, the luciferase activity was measured. All the operations in this experiment followed the manufacturer’s recommendations.

### Western-blotting

Proteins of these cells were lysed and collected with RIPA (Beyotime, China). Next, the concentration of these proteins was determined with the BCA (Beyotime, China) method. Then these proteins were separated with the 10% SDS-PAGE gel (Beyotime, China). Next, the proteins were transferred to the PVDF membranes (Millipore, USA). The PVDF membranes were blocked with the 5% skim milk powder for 1.5 h. Then the membranes were incubated with the primary antibody at 4 °C overnight. The primary antibodies were ROCK1 (#4035, CST), ROCK2 (#9029, CST), Bcl-2 (#4223, CST), Bax (#5023, CST), Cleaved-caspase3 (#9664, CST), Cleaved-caspase9 (#20750, CST), caspase-3(#14220, CST), caspase-9 (#9502, CST), NLRP-3 (#15101, CST), ASC (#13833, CST), Caspase-1 (#24232, CST), IL-1β (#12703, CST), IL-18 (Abcam, ab191152) and p-65 NF-κB (Abcam, ab16502). On the second day, the membranes were washed with PBST three times. Next, the membranes were incubated with the second antibody (Abcam, ab205718). After that the membranes were washed three times with PBST. Final, the membranes were exposed and the band was observed to clarify the changes of expression of these proteins.

### RT-PCR

Total RNA was extracted from the cells by the trizol methods. After that, the mRNA was reverse transcribed into cDNA by the RT reagent Kit (TakaRa, Japan). RT-PCR was performed with the SYBR Green method. The amplification process was performed with the ABI 7500 system (Applied Biosystems, USA). The relative expression of the target genes was calculated by the 2^-∆∆Ct^ method. The following primers were used in this project: miR-136-5p forward primer 5′-ACACTCCAGCTGGGACTCCATTTGTTTT-3′; reverse primer 5′-CCAGTGCAGGGTCCGAGGT-3′; GAPDH forward primer 5′-AATTCCATGGCACCGTCAAG-3′; reverse primer 5′-TGGACTCCACGACGTACTC-3′.

### CCK-8 assay

CCK-8 assays were used to detect cell viability after the stimulation of the LPS. The cells were plated into the 96 well plate. After that, the cells were cultured with the medium containing LPS for 24 h. Then the CCK-8 (Dojindo, Japan) was diluted with the cultured medium and added into the 96 well plate. The 96 well plate was incubated in the incubator for 2 h and finally the absorbance was measured with the microplate Reader.

### ELISA assay

To determine the changes of TNF-α, IL-6 and IL-1β releasing of RPE cells in response to different kinds of stimulation, the levels of TNF-α, IL-6 and IL-1β in the culture supernatant were determined by the ELISA assay. The ELISA kit was obtained from Abcam and Sigma-Aldrich: TNF-α (Abcam, ab181421), IL-6 (Abcam, ab178013) and IL-1β (Sigma-Aldrich, RAB0273). All the operation of this experiment was carried out according to the instructions.

### Apoptosis assay

The different groups of RPE cells were seeded into the six well plates and treated with LPS or ripasudil. After that, these cells were washed with the cold PBS for three times. Then the binding buffer (Beyotime, China) was used to suspend these cells. Next, PI and Annexin V (Beyotime, China) were added into the binding buffer to detect the apoptosis ratios of the RPE cells. The cells were incubated with the PI and Annexin V for 40 min. At last, the apoptosis ratios of these cells were analyzed with the flow cytometry (Beckman, USA).

### Statistical analysis

The analysis of the data in this paper was performed with the GraphPad Prism 7.0. The comparison between different groups was evaluated by the Student’s t test. All the data in this research were presented as mean ± SD and all the experiments in this paper were repeated three times. The difference between diverse groups was considered as statistically significant difference when the value of P was less than 0.05.

## Results

### Ripasudil protected the RPE cells from the LPS induced inflammatory stimulation

**Fig. 1:**
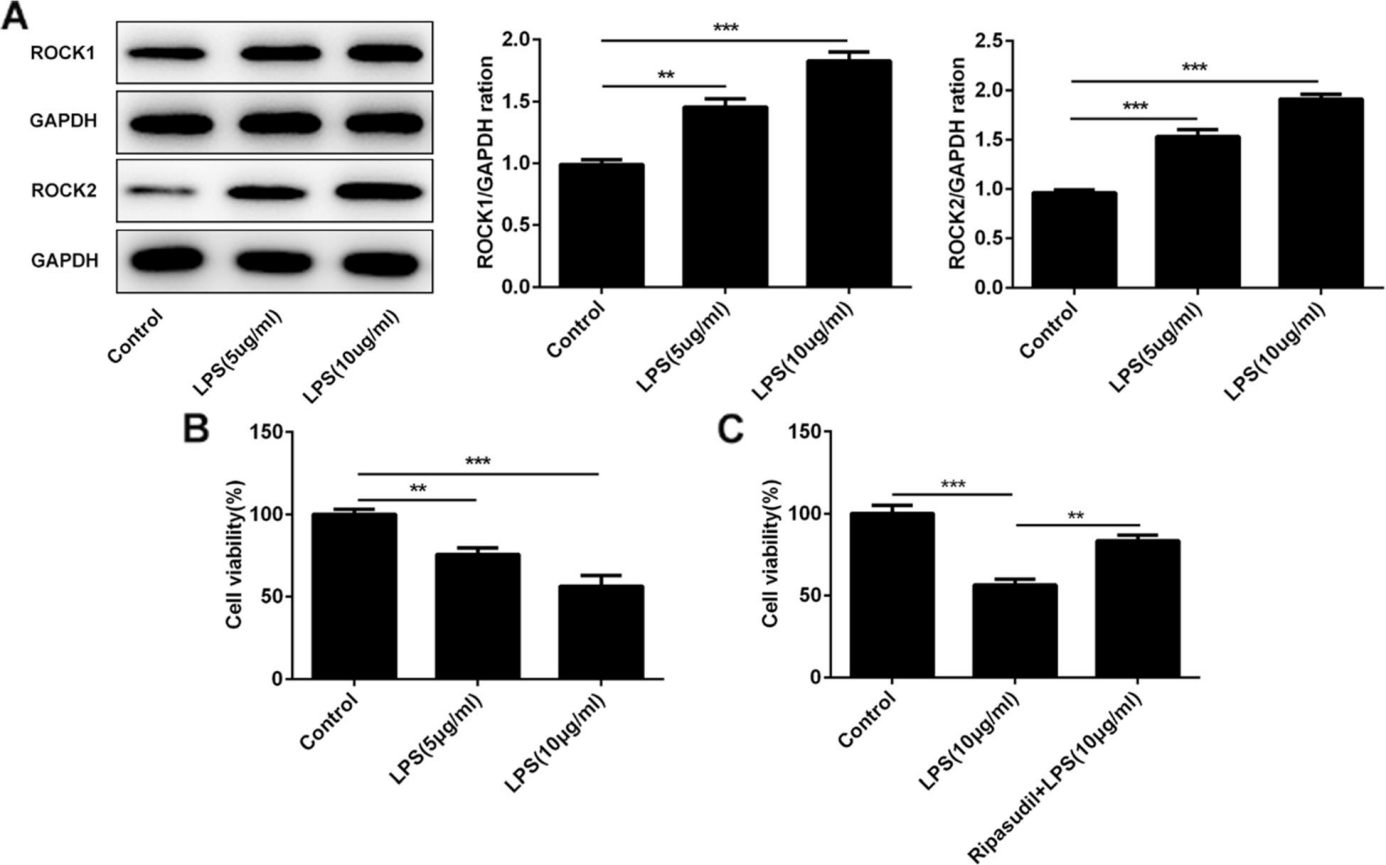
Ripasudil relieved the LPS-induced inflammatory response in RPE cells. **a** Western-blotting was used to determine the expression of ROCK1 and ROCK2 after the stimulation of LPS. **b** The cell viability of RPE cells was detected with the CCK-8 after the stimulation of LPS. **c** The cell viability of RPE cells was determined after the treatment of ripasudil. *p < 0.05, **p < 0.01, ***p < 0.001

LPS was used to stimulate the ARPE-19 cells. After that, the levels of ROCK1 and ROCK2 and the cell viability were determined to evaluate the damage caused by the inflammation. The results (Fig. 1a) showed that the expression of the ROCK1 and ROCK2 was enhanced after the stimulation of the LPS. And the cell viability gradually decreased with the increase of LPS doses (Fig. 1b). Next, ripasudil, the ROCK inhibitor, was used to treat the ARPE-19 cells simulated by the LPS. As shown in Fig. 1c, the cell viability enhanced after the treatment of the ripasudil. All the results in this part indicated that ripasudil could relieve the inflammatory injury induced by LPS.

### Ripasudil relieved the inflammatory damage of RPE cells by targeting miR-136-5p

**Fig. 2:**
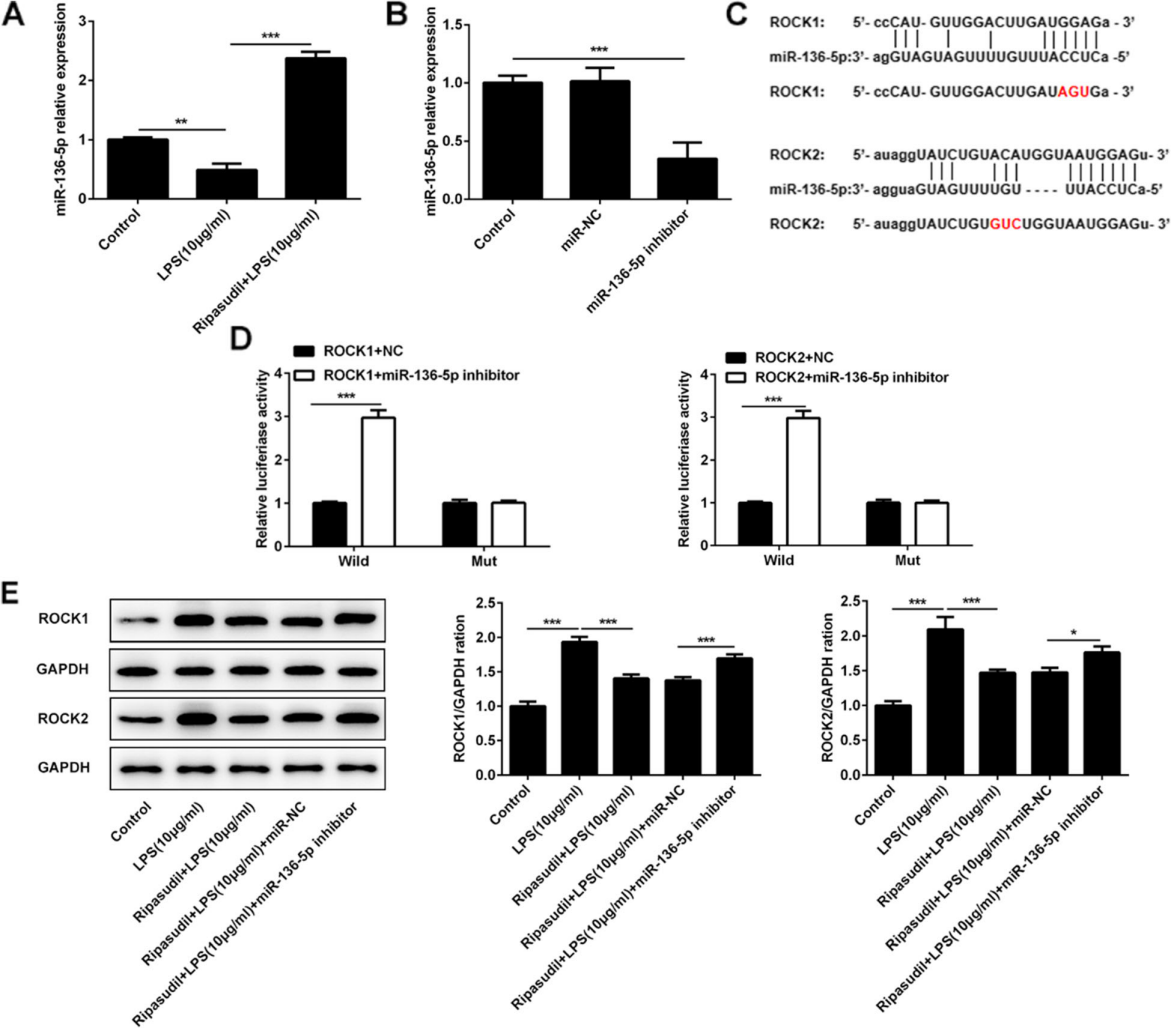
Ripasudil alleviated the inflammatory damage by mediating the expression of miR-136-5p. **a** The relative expression of miR-136-5p in RPE cells was detected with the RT-PCR. **b** The levels of miR-136-5p in RPE cells were determined after the application of miR-136-5p inhibitor. **c** The sequences of mature miR-136-5p and putative target sites of wild type ROCK1 and ROCK2. The mutant sequences of ROCK1 and ROCK2 were marked with red color. **d** Luciferase activity was determined in the miR-136-5p inhibitor and negative control groups. **e** The protein levels of ROCK1 and ROCK2 were detected after the application of miR-136-5p. *p < 0.05, **p < 0.01, ***p < 0.001

One study revealed that miR-136-5p could suppress the expression of ROCK1 [12]. To confirm this notion, in this study, we first used RT-PCR to detect the level of miR-136-5p after the stimulation of LPS and treatment of ripasudil. The results (Fig. 2a) showed that the expression of miR-136-5p was inhibited after the stimulation of LPS. However, the levels of miR-136-5p were upregulated after the treatment of ripasudil. Next, we constructed the miR-136-5p inhibitor. After that, this vector and miR-136-5p was transfected into the RPE cells. The results (Fig. 2b) from RT-PCR showed that the level of miR-136-5p was downregulated in these cells. After that, luciferase reporter assays were performed to evaluate the targeting effect of miR-136-5p on the ROCK1 and ROCK2. To detect the targeted effect of miR-136-5p on ROCK1 and ROCK2, we established the luciferase construct (Fig. 2c) which contained the wild type or mutant of 3′ UTR of ROCK1 and ROCK2. As shown in the Fig. 2d, the reporter assays revealed that the miR-136-5p inhibitor enhanced the luciferase activity, and mutation of miR-136-5p binding site of ROCK1 and ROCK2 3′UTR abrogated the efficacy of miR-136-5p. After that, we determined the level of ROCK1 and ROCK2 after the application of miR-136-5p inhibitor and the treatment of ripasudil or LPS. The results (Fig. 2e) showed that the levels of ROCK1 and ROCK2 were upregulated after the stimulation of LPS, but declined sharply after the treatment of ripasudil. However, the expression of miR-136-5p was rescued in the miR-136-5p inhibitor group compared to the NC group (Fig. 2d).

### Inhibition of miR-136-5p induced the expression of inflammatory factors and apoptosis of RPE cells

**Fig. 3:**
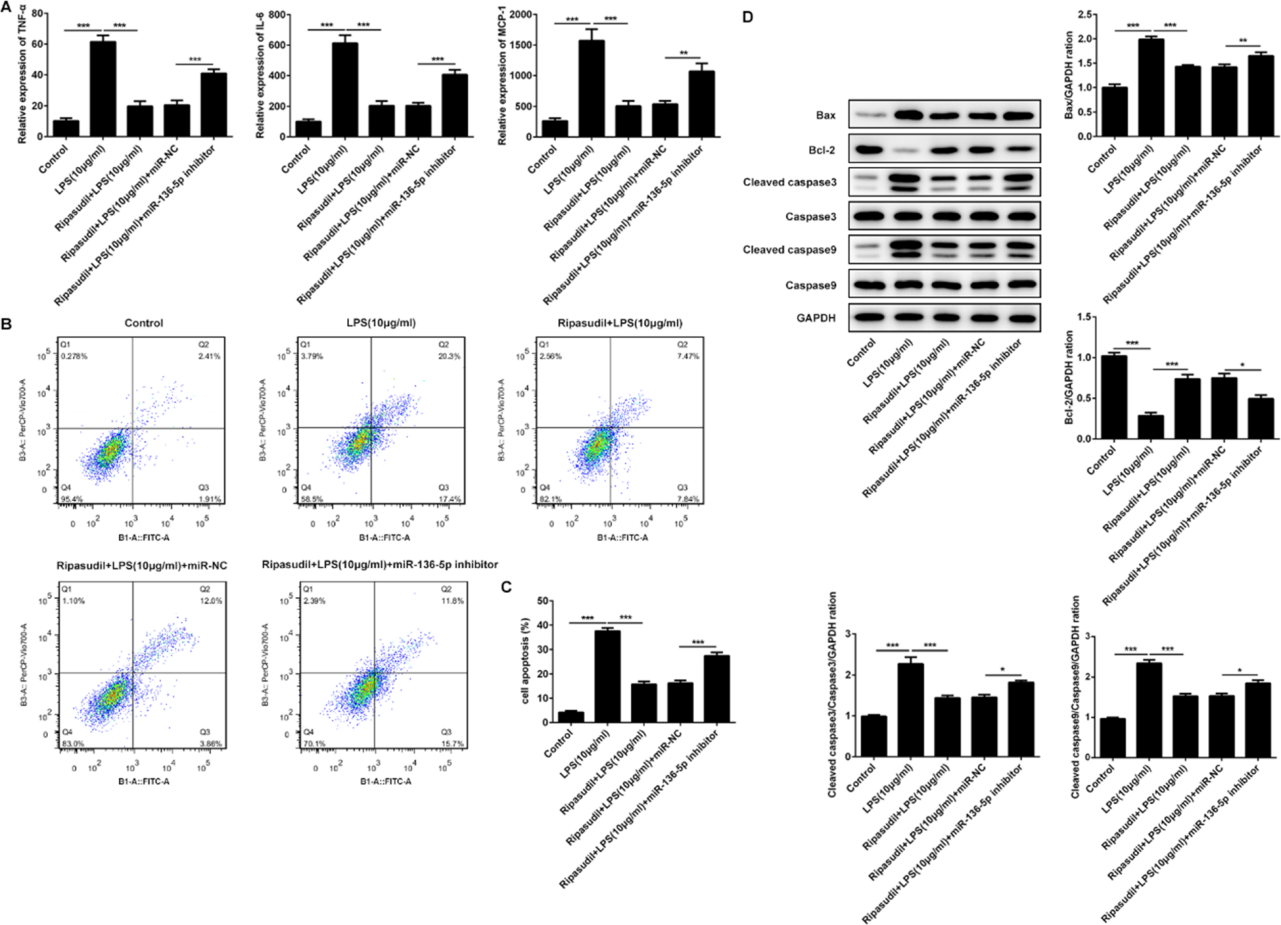
Suppression of miR-136-5p lead to the upregulation of inflammatory factors and increasing apoptosis ratios. **a** The levels of TNF-α, IL-6 and MCP-1 in the supernatant were measured with the ELISA assays. **b** The apoptosis rates of RPE cells were determined with the flow cytometry. **c** The quantitative data of apoptosis rates. **d** The expression of apoptosis-related proteins (Bax, Bcl-2, Caspase3, Cleaved caspase3, Caspase9 and Cleaved caspase9) in RPE cells was detected with western-blotting. *p < 0.05, **p < 0.01, ***p < 0.001

The aberrant upregulation of inflammatory factors was the main reason for the eye diseases, such as diabetic retinopathy and glaucoma. Therefore, ELISA was performed to detect the levels of TNF-α, IL-6 and MCP-1. The results (Fig. 3a) showed that the levels of TNF-α, IL-6 and MCP-1 were increased after the stimulation of LPS but declined after the treatment of ripasudil. After the suppression of the miR-136-5p, the expression of these inflammation factors was rescued. Next, the apoptosis rates of the ARPE-19 cells was detected to explore the effects of ripasudil and miR-136-5p on these cells which were stimulated by the LPS. As shown in Fig. 3b and Fig. 3c, the ripasudil relieved the apoptosis which was induced by the LPS, whereas the inhibition of the miR-136-5p aggravated the apoptosis of ARPE-19 cells. At last, the expression of the apoptosis-related proteins was determined by the western-blotting. The results (Fig. 3d) showed that the levels of the Bcl-2 were decreased while the expression of Bax, cleaved-caspase3 and cleaved-caspase9 was inhibited after the treatment of ripasudil. However, after the inhibition of miR-136-5p, the levels of Bax, cleaved-caspase3 and cleaved-caspase9 were enhanced while the expression of Bcl-2 was repressed.

### Ripasudil inhibited the inflammatory response by targeting NLRP3/ASC/caspase-1 pathway

**Fig. 4:**
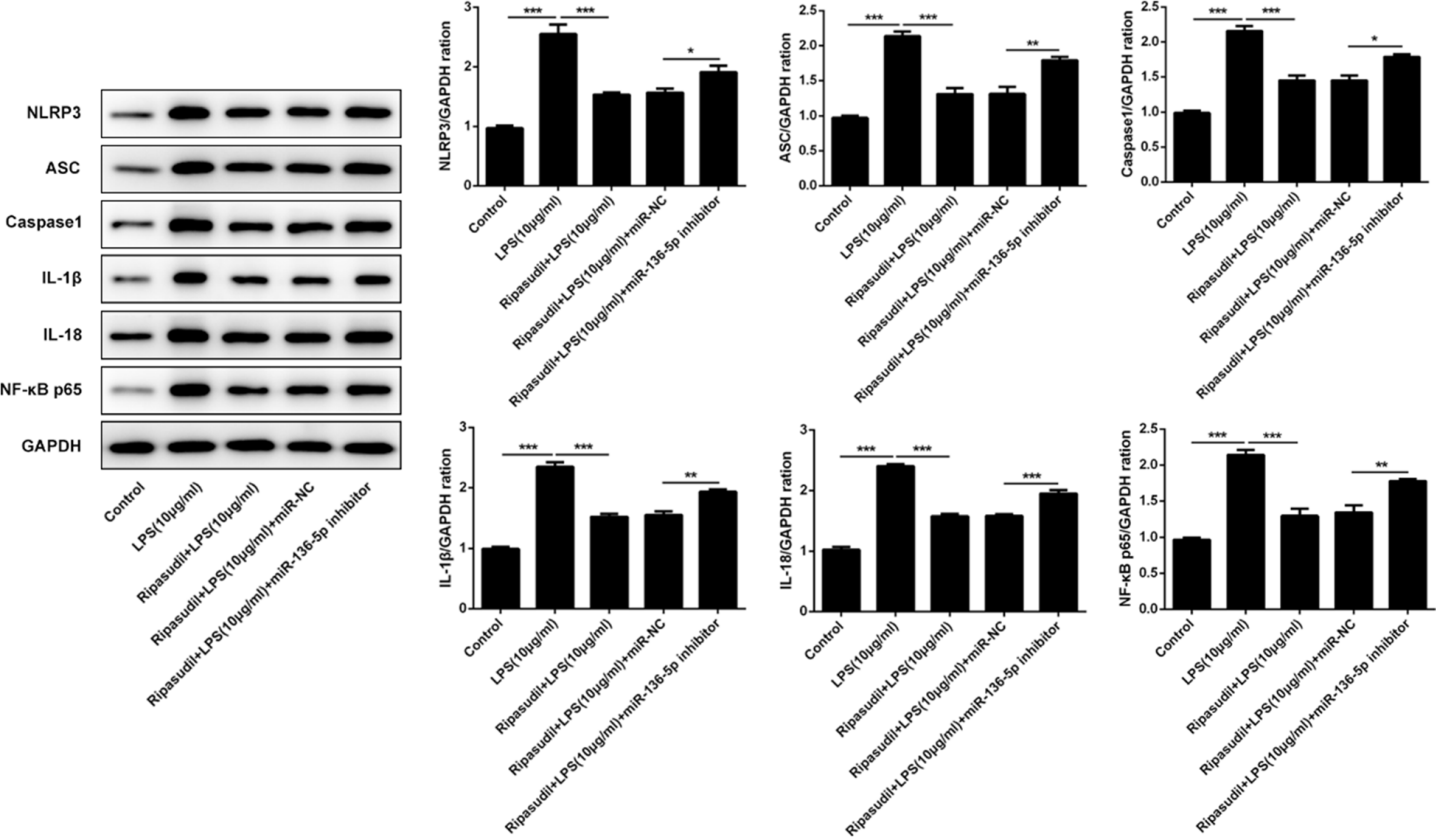
Ripasudil relieved the inflammation induced damage of RPE cells by suppressing the NLRP3/ASC/caspase-1 pathway. The levels of NLRP3, ASC, Caspase1, IL-1β, IL-18 and NF-κB in RPE cells were determined with the western-blotting. The quantitative of these proteins was performed with ImageJ. *p < 0.05, **p < 0.01, ***p < 0.001

The NLRP3/ASC/pro-caspase1 was a protein complex that played a critical role in the inflammatory process [14]. The formation of this complex led to the activation of the caspase-1, thereby inducing the expression of IL-1β and IL-18. Therefore, western-blotting was performed to detect these proteins. As shown in Fig. 4, the expression of NLRP3, ASC, caspase-1, IL1β, IL-18 and NF-κB p65 was inhibited after the treatment of ripasudil. Nevertheless, the levels of these proteins partially were recovered after the inhibition of the miR-136-5p.

## Discussion

Inflammation was a potential cause of many eye diseases, including age-related macular degeneration (AMD), autoimmune uveitis, glaucoma and diabetic retinopathy [15]. RPE cells were invaded by various inflammatory mediators and infectious factors because of their crucial position and physiological activity [16]. The inflammation of the RPE cells could finally lead to the occurrence and development of the AMD. AMD was the leading cause for the loss of vision in older people group [17]. On the other hand, ROCK1 and ROCK2 played a critical role in regulating endothelial function. It was clear that the ROCK pathway could lead to the dysfunction of endothelium by activating the NF-κB, therefore the higher levels of ROCK1 and ROCK2 were associated with the occurrence and development of inflammation injury [18, 19]. However, ripasudil which was used as a drug for the treatment of glaucoma in Japan was also one of the inhibitors of ROCK1 and ROCK2 [20]. And some research revealed that the ripasudil could alleviate the LPS-induced inflammation and apoptosis of pulmonary microvascular endothelial cells [11]. Furthermore, the study also showed that the ripasudil hydrochloride hydrate could relieve the inflammation and intraocular hypertension [21]. In our study, we found that the stimulation of LPS led to the upregulation of ROCK1 and ROCK2 while the treatment of ripasudil inhibited the expression of TNF-α, MCP-1, IL-6, ROCK1 and ROCK2. Meanwhile, treatment with ripasudil also decreased the apoptosis rates and increased cell viability of these cells. These results indicated that the ripasudil protected the RPE cells from the LPS-induced inflammation. The results also suggested that the ripasudil maybe protected the RPE cells from inflammation by targeting ROCK1 and ROCK2. Therefore, we designed the experiments to further detect the mechanism of ripasudil therapy.

Moreover, there is research revealed that miR-136-5p suppressed the inflammatory response which was induced by the cerebral ischemia/reperfusion [12]. The prediction results of targetscan showed that miR-136-5p may target and regulate the expression of ROCK1 and ROCK2. In this study, we found that the miR-136-5p targeted the promoter region of ROCK1 and ROCK2. The expression of ROCK1 and ROCK2 was promoted after the inhibition of miR-136-5p. Furthermore, the levels of miR-136-5p were downregulated after the stimulation of LPS and the expression of miR-136-5p was rescued after the treatment of ripasudil. These results indicated that the ripasudil relieved the inflammation damage by upregulating of the miR-136-5p and the higher levels of miR-136-5p targeted and suppressed the levels of ROCK1 and ROCK2.

The NLRP3/ASC/pro-caspase1 was a protein complex that came into being during the process of inflammatory response [14]. The formation of this complex would lead to the production of active caspase-1. After that the active caspase-1 could lead to the upregulation of IL-1β and IL-18. Finally, the higher levels of IL-1β and IL-18 induced the inflammatory response and apoptosis of cells [14, 22, 23]. Furthermore, there is research revealed that the inhibition of ROCK1 and ROCK2 greatly restricted the expression of NLRP3 and therefore suppressed the inflammation which was induced by the higher levels of NLRP3 [24]. In our study, we discovered that the treatment of ripasudil relieved the upregulation of NLRP3, ASC, caspase1, IL-1β and IL-18. After the suppression of miR-136-5p, the levels of these proteins were rescued. Therefore, all the results in this paper implied that the treatment of ripasudil alleviated the inflammation damage of RPE cells by enhancing the level of miR-136-5p, therefore inhibiting the expression of ROCK1, ROCK2, NLRP3, ASC, caspase1, IL-1β and IL-18. Finally, the release of inflammatory factors declined and the inflammatory injury was relieved. Our study also provided the new approach and target for the treatment of eye diseases, which were caused by the inflammation of RPE cells.

## Conclusion

Above all, in this study we revealed that the application of ripasudil alleviated the inflammatory injury of RPE cells by upregulating miR-136-5p and suppressing the expression of ROCK1, ROCK2, NLRP3, ASC and caspase1. And this research could provide the new target for the clinic treatment of the inflammation of RPE cells.

## Data Availability

The analytical data in this study could be obtained from the corresponding author upon reasonable request.
